# Anti-cancer activity of *C. cainito* leaves explained using its compounds docked with p53

**DOI:** 10.6026/97320630018870

**Published:** 2022-10-31

**Authors:** Yesudass Antony Prabhu, Chinnasamy Balalakshmi, Muthu Vijayasarathy, Kumar Praveen Kumar, Piramanayagam Shanmughavel, Samiappan Kavitha

**Affiliations:** 1 Department of Biochemistry, Rathnavel Subramaniam College of Arts and Science, Bharathiar University, Coimbatore - 641402, India; 2 Department of Nanoscience and Technology, Alagappa University, Karaikudi - 630003, India; 3 Department of Microbiology, Thiagarajar College of Arts and Science, Madurai, India; 4 Department of Bioinformatics, Bharathiar University, Coimbatore - 641046, India; 5 Department of Bioinformatics, Bharathiar University, Coimbatore - 641046, India; 6 Department of Biochemistry, Rathnavel Subramaniam College of Arts and Science, Bharathiar University, Coimbatore - 641402, India

**Keywords:** molecular docking, mutant P53 inhibitors, *C. cainito*, OSCC

## Abstract

Extensive research on the mutant P53 protein has identified its pivotal role in anti-apoptosis mechanisms, drug resistance, and cancer progression in OSCC. The mass spectrum revealed the pharmacologically significant bioactive compounds reported for the first time in C cainito. Molecular docking investigation has identified four potential new P53 inhibitors compared with the standard P53 inhibitors. Hence, this analysis reinforces the likelihood of anti-cancer activities in *C. cainito* leaves.

## Background:

OSCC is a heterogeneous group of cancers in the oral cavity, accounting for about 90% of all oral malignancies [1-2]. The traditional risk factors associated with OSCC include smoking habits with a cigarette, bidis, kretek or water pipe; chewing habits with betel squid, pan masala, and consumption of excessive alcohol [3]. Most significantly, the habits of tobacco smoking and alcohol consumption potentiate the induction of p53 mutations in nearly 65-85% of OSCC patients [4]. Generally, wild-type p53 mediates cell cycle arrest by p21 activation, regulation of DNA repair, and apoptosis induction which acts as a tumor suppressor gene and prevents OSCC progression. Conversely, a mutation in p53 leads to the gain of proto-oncogenic function, thereby increasing the progression of tumorigenesis and carcinogenesis. Thus, p53 mutants have become a popular target for developing new cancer therapeutics [5]. Furthermore, patients who underwent cancer therapy, such as chemotherapy and radiation therapy, have developed drug resistance, promoting tumor progression, and poor survival, and are associated with the p53 mutation [6]. Recent research has uncovered that alteration of the p53-encoding gene TP53 is the most direct and effective route to inactivate p53 [7]. Consequently, it is difficult to cure cancer, and the incidence of OSCC has expected to be more in the future in India [2]. Intrinsically, such mutations interfere with anti-apoptotic mechanisms in the OSCC. Hence, it is of interest to document the effective anti-mutant p53 inhibitors from *C cainito* leaves using molecular docking.

## Materials and methods:

### Plant collection:

The fresh and healthy leaves of C cainito (latitude 11.01° N, longitude: 76.9 5° E) were collected with fruit specimens and submitted for authenticity at the Botanical Survey of India, Tamilnadu Agricultural University campus, Coimbatore. Subsequently, we received for voucher number of the analyzed specimen as BSI/SRC/5/23/2020/Tech/808.

### Soxhlet extraction of plant material:

At the onset, *C cainito* leaves were washed three times with ultra-pure Milli Q water to clean off impurities and soil. After drying them at room temperature for a week, it was well pulverized and stored in an airtight jar until needed. For soxhlet extraction, 100 grams of *C cainito* leaves powder were subjected to ethanolic extractions using the soxhlet apparatus at 60°C for 12 hours. In this extraction, HPLC grade of solvent was used. Subsequently, the concentrated ethanolic sample was kept at 50°C for two days in a hot air oven on a washed petri plate and analyzed using GCMS.

### Phytochemical analysis using GC-MS:

The bioactive compounds presence in the ethanolic extract of the C cainito specimen had explored by Thermo MS DSQ II, Thermo Fisher Scientific, United States. For a sample running GCMS conditions, refer to our journal [2]. The analyzed chromatogram of ethanolic extract of *C cainito* is shown in Figure 1(see PDF).

### Molecular Docking:

Molecular docking analysis approach was performed using the maestro module of the Schrodinger suite because of its intuitive molecular modelling environment-like features for medicament discovery. The Glide module of the XP visualizer was used with the OPLS-2005 force field to identify an interaction between C cainito ligands and altered p53 protein binding sites, as well as their affinity [2].

### Preparation of protein:

The 3-dimensional molecular structure of a mutant P53 protein (6GGE) is obtained from PDB website (website link: https://www.rcsb.org/). The 6GGE protein was prepared using the protein preparation wizard tool (prep wizard) in Schrodinger Suite, 2018. Prep wizard is an indispensable tool for researchers to correct the structural imperfections [8-9]. Figure 2(see PDF) illustrates the massive disparities between unprocessed and preprocessed 6GGE structures. The unprocessed structure of 6GGE was found to hold faults, whereas the preprocessed protein structure is subjected to a prep wizard. All 3D structures of C cainito phytonutrients were retrieved from the PubChem database and created as ligands using the Lig Prep module in the same Schrodinger suite using GCMS results [10]. However, the computational investigations require the following features like default parameters: 3D structures conversion, precise lewis chemistry, stereoisomers, ring conformations, limits the computational errors, and customizable libraries. Phytonutrients analyzed from an ethanolic extract of *C cainito* are listed in Table 1(see PDF) and whereas Figure 3(see PDF) illustrates the structures of all the investigated chemical compounds.

### Analysis of ADME/T property:

Several medicaments failed nearly 40% in clinical trials due to poor ADME characteristics. Late-stage failures are a major contributor to the rising expense of new therapeutic development. The QikProp tool predicts the properties of molecules with new scaffolds and analogs of well-known medications with comparable accuracy. By which, the ADME/T characteristics of the best four interacted ligands over 6GGE were assessed using the QikProp module in the Schrodinger suite. QikProp investigation exposed significant physicochemical characteristics and pharmacokinetic relevant features (Table 2 - see PDF) [2].

## Results and Discussion:

It is the first investigation which is performed from ethanolic extract of C cainito leaves against mutant P53. Thereof, MACA interacts with 6GGE molecules as shown in Figure 4 A and B(see PDF). Ligand formed a strong hydrogen bond with the mutant p53 receptor amino acid residues at arginine 196 and asparagine 186 during this interaction. These molecules have bond distances of 2.18, 2.42, and 1.89, respectively. The top four ligands that interacted with the mutant P53 receptor (as indicated in Table 3 - see PDF) had docking GS scores of -6.08, -4.63, -4.45, and -4.29, respectively. These ligands include MACA, 2-hydroxyacetamide, 2-phenylprophylamine, and dihydroxymalonic acid. A greater negative GScore has a better interaction [11] and Table 3(see PDF) also lists other metrics such as dock score and H bond. Table 4(see PDF), the ligand-interacting amino acid residues and bond lengths of the molecules are listed. This study uncovered a unique MACA interaction that is expected to have the same function and anti-cancer properties. For instance, molecular docking research by Hussan and colleagues [12] showed that benzalkonium ibuprofenate interacted better than built-in standard ligands with a receptor. In a similar fashion, we have sought to validate such feasibility with a commonly known two standard p53 inhibitors, such as ellipticine and pifithrin-α p-nitro [13-14]. Surprisingly, we found that our four ligands are mentioned above interacted more strongly with mutant p53 than the standard p53. The pifithrin-α p-nitro had interacted slightly effectively as compared with ellipticine, at the docking scores of -3.81 and -3.76. This evidence reinforces the in-silico approach and the prospect of finding novel mutant p53 inhibitors from the medicinal plant C cainito (Figure 5 and Table 3 - see PDF). Next to that MACA inhibitor, the other three ligand interactions at binding sites, bond type, and length, as well as providing GScore, are shown in Figure 5(see PDF). Furthermore, ADMET characteristics of bioactive compounds discovered in the ethanolic extract of C cainito are reported in Table 2(see PDF) MACA and 2-phenylprophylamine have good properties.

## Conclusions:

Data reports the potent four effective mutant P53 inhibitors from the C cainito leaves, which hold better binding properties than ellipticine and pifithrin-α p-nitro. With the highest docking score over the mutant P53 receptor as compared to 2-hydroxyacetamide, 2-phenylprophylamine, and dihydroxymalonic acid, MACA inhibitors are the best ligands for interactions. However, further analysis of ADME/T characteristics demonstrated the drug-likeliness for 2-phenylprophylamine and MACA bioactive compounds. On the other side, 2-hydroxyacetamide and dihydroxymalonic acid had minimal pharmacokinetic properties. Therefore, we suggest that MACA and 2-phenylpropylamine are novel mutant P53 inhibitors that deserve augmented attention as anti-cancer agents.
